# Fertility Preservation in Female Pediatric Patients With Cancer: A Clinical and Regulatory Issue

**DOI:** 10.3389/fonc.2021.641450

**Published:** 2021-03-09

**Authors:** Serena Brancati, Lucia Gozzo, Laura Longo, Daniela Cristina Vitale, Giovanna Russo, Filippo Drago

**Affiliations:** ^1^ Clinical Pharmacology Unit/Regional Pharmacovigilance Centre, University Hospital of Catania, Catania, Italy; ^2^ Department of Biomedical and Biotechnological Sciences, University of Catania, Catania, Italy; ^3^ Pediatric Onco-Hematology, University Hospital of Catania, Catania, Italy; ^4^ Centre for Research and Consultancy in HTA and drug Regulatory Affairs (CERD), University of Catania, Catania, Italy

**Keywords:** GnRHa, chemotherapy, adverse event, off-label, regulatory issue

## Abstract

Fertility preservation represents one important goal of cancer patients’ management due to the high impact on health and quality of life of survivors. The available preventive measures cannot be performed in all patients and are not feasible in all health-care facilities. Therefore, the pharmacological treatment with GnRHa has become a valuable non-invasive and well-tolerated alternative, especially in those who cannot access to cryopreservation options due to clinical and/or logistic issues. Supporting data demonstrate a significant advantage for the survivors who received GnRHa in the long-term maintenance of ovarian function and preservation of fertility. The prevention of the risk of ovarian failure with GnRHa is a typical off-label use, defined as the administration of a medicinal product not in accordance with the authorized product information. Italy has officially recognized the off-label use of GnRHa in adult women at risk of premature and permanent menopause following chemotherapy. However, fertility preservation still represents an unmet medical need in adolescents who cannot access to other treatment options.

## Introduction

In Europe, nearly 80% of children and adolescents with cancer treated on current protocols survive at least 5 years on average (1). Improved survival rates have increased the number of childhood cancer survivors (CCSs) entering adulthood after treatment for malignancy, which account for 0.1–0.15% of the general population (2). The high survival rate in children and adolescents is accompanied by a substantial risk of late adverse events (LAEs). Above all, treatment may interfere with physiological growth and development and have an important impact on health status later in life, whilst some late toxicities may cause premature death. Fertility is one of the most important concerns of CCSs (3, 4). The occurrence of fertility impairment in female—reduced pregnancy rates and increased risk of early menopause—after pelvic, abdominal, or spinal radiotherapy, total body irradiation, or chemotherapy regimens containing alkylant agents during childhood and adolescence has been widely documented (5–8).

Treatments may deplete or accelerate the decline of the non-renewable pool of primordial follicles in the ovary leading to POF and infertility (9, 10). Gonadal toxicity is affected by type, doses and length of therapy (11, 12), and by age at treatment (females treated at a younger age are less likely to develop POF, probably because of a higher number of primordial follicles at the time of treatment). POF results in a reduced fertile lifespan and associated risk for involuntary childlessness, which can negatively impact the quality of life (13–15), but also accelerates the risk of developing menopause-associated conditions, such as osteoporosis and cardiovascular disease (16). Therefore, fertility preservation (FP) represents an important issue for oncologists, fertility specialists and patients. Most recent guidelines (12, 17, 18) state FP should be discussed with the parents (or guardians) of adolescents soon after diagnosis and before starting anticancer treatments.

In pubertal and postpubertal adolescents, oocyte cryopreservation is one of the available option for FP during gonadotoxic chemotherapy (18) (**Table 1**). Oocyte cryopreservation (21) requires ovarian stimulation with gonadotropin hormone, ultrasound-assisted oocyte collection, and selection and freezing of oocytes (22). There are many barriers to the adoption of this option into standard of care. First, oocyte cryopreservation should be carried out before starting chemotherapy and requires time (17) for ovarian stimulation and follicular growing, with consequent delay in the initiation of oncological treatment. This can be a problem especially in pediatric cancers, which often require urgency to start treatment. The random-start controlled ovarian stimulation protocol, providing for a stimulation at any time of the menstrual cycle, can reduce the time needed for cryopreservation, but for patients with very aggressive diseases no delay is allowed (23). Moreover, this technique is associated with additional challenges as it requires to acquire oocytes through transvaginal approach (24), a painful procedure to be performed with sedation and with specialized equipment, which is not often available in pediatric hospitals. Not less important is its emotional and psychological impact for most adolescents, despite their sexual maturity.

**Table 1:** Treatment options for fertility preservation.

Technique	Definition	Advantages	Disadvantages	Experimental
Oocyte cryopreservation	Controlled ovarian stimulation, followed by oocyte retrieval and cryoconservation for future use	- well-established fertility preservation technique - no ethical issues	- time required for ovarian stimulation - risk of overstimulation - invasive procedure for oocyte retrieval (day surgery) - not recommended in women with hormone-sensitive cancers - not possible for prepubertal girls - need of a male partner/donor for oocyte fertilization prior to implantation - high cost	No
Embryo cryopreservation	Controlled ovarian stimulation, followed by oocyte retrieval, in vitro fertilization and embryo cryopreservation for future use (19, 20).	- well-established fertility preservation technique - good embryo survival to thawing - direct transfer into the uterus after thawing	- ethical issues regarding embryo disposition - time required for ovarian stimulation and subsequent delay in timely cancer treatment - risk of overstimulation - invasive procedure for oocyte retrieval (day surgery) - not recommended in women with hormone-sensitive cancers - not possible for prepubertal girls - need of a male partner/donor - high cost	No*
Ovarian Tissue cryopreservation	Surgical retrieval of ovarian tissue, cryopreservation of the tissue and subsequent reimplantation once patient is disease-free	- feasible for prepubertal children - does not require hormonal stimulation - can be planned shortly after diagnosis of malignant disease	- surgical procedure under general anesthesia - risk of the re-introduction of carcinogenic cells - risk of malignant transformation of the ovarian tissue - risk of ischemic damage to the tissue - limited availability of centers with adequate cryoconservation competences and able to perform the most sensitive and updated histological analysis techniques before transplantation to avoid relapses - high cost	Yes
Ovarian suppression with GnRHa	Concomitant use of gonadotropin-releasing hormone analogs (triptorelin, goserelin, leuprolide) during the course of chemotherapy to induce a prepubertal hormonal milieu and preserve the ovarian function	- not invasive - does not need delaying in oncologic therapy - can be used in association with cryopreservation techniques - reduce the risk of hypermenorrhea associated with hematologic malignancies or myelosuppressive treatments - low cost	- symptoms of estrogenic deprivation - transient alterations of bone metabolism not significant for therapy duration < 6 months - limited clinical evidences in patients with disease other than breast cancer	Yes**

*Forbidden in Italy (Law 40/2003); **Reimbursed in Italy for oncologic adult patients according to Law 648/96.

Ovarian tissue cryopreservation (OTC), although still considered as experimental, is commonly proposed as an alternative to oocyte cryopreservation (18). Cryopreservation of the ovarian tissue requires a laparoscopic procedure under general anesthesia and the subsequent freezing of the tissue that contains most primordial follicles (25). Differently from oocyte cryopreservation, OTC can be performed at any time with less delay in starting cancer therapy. Once the patient is disease-free, an autotransplantation can be carried out. Major issues of OTC include ischemic damage to the tissue and the theoretical risk of reintroducing malignant cells, especially. Indeed, transplantation is not in patients with diseases associated with a high risk of ovarian metastases (26, 27). Another limitation is represented by the availability of a center with adequate cryoconservation competences and able to perform the most sensitive and updated histological analysis before transplantation to avoid relapses (28).

An alternative, that could represent a more accessible FP option burdened by less discomfort for postpubertal patients, could be the concomitant use of gonadotropin-releasing hormone (GnRH) analogs (triptorelin, goserelin, leuprolide) during the course of chemotherapy (18, 29, 30). This non-invasive and less expensive approach could allow preventing POF in cancer survivors and has been recently integrated in clinical guidelines (17, 18, 31–34). In particular, the updated European Society for Medical Oncology (ESMO) and American Society of Clinical Oncology (ASCO) recommendations state that GnRHa should be used in addition to the other options (17, 35).

It is noteworthy that in adolescents and young women with malignancies GnRHa are widely used as an alternative to estro-progestinic combinations for menstrual suppression (36–41), in order to reduce the risk of heavy menstrual bleeding associated with hematologic malignancies or myelosuppression induced by chemotherapy. In this setting, despite significant side-effects simulating the physiology of menopause and the risk of loss of bone mineral density with prolonged use (usually > 6 months) (42), GnRHa are well tolerated and effective option for menses suppression (43, 44) and are generally preferred to oral contraceptives for several reasons. Oral contraceptives have some disadvantages such as the daily regimen, in contrast to the monthly administration of GnRHa. Furthermore, the efficacy of oral contraceptives can be reduced by an erratic absorption due to mucositis, diarrhea, and emesis. Moreover, the use of estrogen-based oral contraceptives for menstrual suppression is associated with an increased risk of venous thromboembolism (41, 45). However, one of the most important reason for choosing GnRHa is represented by their possible gonadal protective effect (41).

## The Clinical Perspective: Concluded and Ongoing Trials

The rational for the use of GnRH analogs (GnRHa) for the reduction of ovarian toxicity is based on the observation that chemotherapy mostly affects tissues with rapid cellular turn-over, as gonadal one (46). Moreover, the gonadotoxicity is lower in prepubertal girls than in adult women (47, 48), probably as a consequence of their higher ovarian reserve, in addition to the hypogonadotropic prepubertal milieu. This led to the speculation that ovarian suppression in postpubertal female patients might mitigate the adverse effects of treatment on ovarian function (49). Preclinical data have confirmed the efficacy of GnRHa in reducing cyclophosphamide-induced gonadotoxicity (50–53). A prospective randomized study in primates demonstrated that co-treatment with GnRHa significantly decreased the rate of follicular decline and the total number of primordial follicles lost compared with cyclophosphamide alone (53).

GnRHa may protect the ovaries against chemotherapy-induced damage through the inhibition of the hypothalamic-pituitary-ovarian axis with the induction of a prepubertal state (54). The increased rate of non-resting follicles loss leads to a decrease in the secretion of sex steroids and inhibins produced by these follicles at different stages of maturation and differentiation. The resultant low systemic concentrations of these endogen molecules induce a feedback on the hypothalamus and pituitary gland, increasing the gonadotropins secretion, mainly follicle-stimulating hormone (FSH) (54), which enhance the follicle recruitment and maturation. These growing follicles are more exposed to the gonadotoxic effects, ending in an accelerated rate of follicular apoptosis and degeneration. This vicious cycle may be interrupted by preventing the increase in FSH through the GnRHa administration (55). Moreover, GnRHa may exert beneficial effects through the decrease in utero-ovarian perfusion resulting from the hypoestrogenic state (56, 57), with lower exposure of the ovaries to chemotherapy. In addition, human gonads express GnRH receptors (58–63), which activation may decrease apoptosis (61). Other hypothetical gonadoprotective mechanisms include an increased formation of intragonadal antiapoptotic molecules (64, 65) and protection of the undifferentiated germline stem cells (54), with the latter mechanism not yet tested under an experimental model.

Several randomized clinical trials (RCTs) have shown a clear benefit and a good safety profile of GnRHa in the prevention of chemotherapy damage in pre-menopausal women aged 18–45 years with breast cancer (66–76) (**Supplementary 1**). In these RCTs, patients with normal ovarian function were randomized to receive chemotherapy plus GnRHa or alone. The study population was heterogeneous, with different age, chemotherapy regimens, selection criteria and follow-up duration. The markers for FP were mainly represented by the return of ovarian function, and the assessment of ovarian reserve by measurement of hormone levels. Only few studies evaluated the long-term pregnancy rate in survivors, the most appropriate marker of fertility that requires a prolonged follow-up, especially in a young population. The three larger phase III RCTs [US POEMS–SWOG/S0230 (73, 74), Italian PROMISE–GIM6 (68) and Anglo Celtic Group OPTION trial (75)], demonstrated a statistically significant reduction in POF and a significant increase in pregnancy rate in the GnRHa arms. A large meta-analysis of 12 RCTs including patients with breast cancer (77) confirmed these positive findings showing a significant reduction of the risk of POF at 12 months from the end of chemotherapy, and a greater number of pregnancies. The efficacy and safety of GnRHa as a clinical option to reduce POF and improve fertility was further confirmed by a recent meta-analysis and systematic review (78), that showed a statistically greater number of pregnancies in the GnRHa group and no differences in progression-free survival.

The similar or even improved survival outcomes of premenopausal breast cancer patients who received GnRHa, reported by the main RCTs and meta-analysis (79) dispelled the safety concern on the potential antagonism between concurrent GnRHa and chemotherapy.

Moreover, no significant increase in the occurrence of GnRHa-associated toxicities (e.g. hot flashes, sweating, headache, vaginal dryness, and thromboembolic events) has been reported (68, 73).

In the light of these results, the most updated guidelines consider temporary ovarian suppression with GnRHa during chemotherapy as an option to be discussed with breast cancer patients interested in preserving ovarian function (17, 80, 81).

Currently, limited evidence exists on the role of this strategy in women diagnosed with tumors other than breast cancer. One randomized trial has assessed the temporary ovarian suppression with GnRHa in 30 young patients with ovarian cancer (82). The study showed a significant reduction in the risk of chemotherapy-induced POF, although no information on post-treatment pregnancies was reported.

Randomized trials performed in women with hematological malignancies showed no protective effect of GnRHa or suggested a partial protective effect with only a delaying in the appearance of POF (83–86). It is noteworthy that all these studies had a small sample size and were not powered to find a possible advantage of GnRHa. Indeed, when the gonadotoxicity is either very low or very high (>90%) the needed power to detect a difference between the study arms requires hundreds of patients. However, other large retrospective or prospective studies and case series have shown a potential protective effect of GnRHa during chemotherapy also in women with hematological malignancies (87–97).

Nevertheless, at present, only very limited evidences from RCTs regarding the use of GnRHa in adolescents with cancer are available (**Table 2**, **Supplementary 2**). This could be due, at least in part, to the difficulty of carrying out, in such a young population, clinical trials with a follow-up long enough to allow the evaluation of reliable fertility preservation indicators, such as pregnancy rate. Indeed, menses resumption is an indirect marker of fertility, but patients resuming menses may have a subclinical and irreversible depletion of ovarian reserve and may experience early menopause (102, 103). The only prospective phase III RCT including postmenarchal adolescent patients, affected by ovarian malignancy, demonstrated the gonadoprotective effect of GnRHa even in the younger population (82). Six months after chemotherapy, all the patients in the GnRHa group had normal menstrual bleeding and normal titer of FSH/LH, whereas 33% in the control group had amenorrhea and POF. A phase II trial evaluated the gonadoprotective effect of leuprolide in adolescent and young women affected by hematologic malignancies who underwent to hematopoietic stem cell transplantation (HSCT) (98). In this case only seven patients (16%) regained ovarian function and leuprolide failed to significantly preserve fertility.

**Table 2:** Studies involving adolescent patients.

Author (Study Design)	Number of Patients(Disease)	Age range(years)	Chemotherapy protocol	GnRHa (dosage/posology-duration)	Treatment arms (patients per arm)	Outcome measures	Follow-up duration
Gilani et al. (82) (Phase III, randomized clinical trial)	30 (Ovarian malignancies)	12–40	Three to seven courses of one of the following regimens: VAC; BEP; TC; CP	Triptorelin depot, 3.75 mg, i.m., administered 7 days before starting chemotherapy and every 28 days during chemotherapy treatment	1. Chemotherapy + triptorelin (15) 2. Chemotherapy alone (15)	Resumption of menses and serum FSH level < 20 mIU/ml at 6 months post- chemotherapy	6 months
Cheng et al. (98) (Phase II, open label, non-randomized clinical trial)	60 (Hematologic malignancies, mainly HD and AML)	15–39	HSCT myeloablative (cyclophosphamide+TBI; etoposide+TBI; busulfan+ cyclophosphamide; busulfan+ melphalan; busulfan+ fludarabine; carmustine, etoposide, cytarabine, and melphalan) or non-myeloablative (fludarabine+melphalan; fludarabine+cyclophosphamide; cyclophosphamide; melphalan+arsenic) conditioning regimens	Leuprolide 22.5 mg in a 3-month depot i.m. injection, given within 2 months before stem cell transplantation. The second dose of leuprolide was given 3 months after the first injection.	1. Leuprolide + HSCT conditioning regimen (60)	Resumption of menses and monitoring of FSH, LH, and estradiol levels	355 days (median; range 102–1,676 days)
Castelo-Branco et al. (89) (Open label, comparative, non-randomized, prospective study)	56 (HD)	14–45	C-MOPP-ABV; C-MOPP-ABV+RTP; C-MOPP-ABV+MINE-ESHAP+ASCT; C-MOPP+ABVD; ABVD; ABVD+RTP; ABVD+MINE-ESHAP+ASCT; ABVD+RTP+MINE+ESHAP+ ASCT; C-MOPP/ABV+RTP+ ASCT; ABVD+C-MOPP+RTP	Triptorelin depot, 3.75 mg, i.m., 1–2 weeks before starting chemotherapy and every 4 weeks during chemotherapy treatment + 2.5 mg daily tibolone	1. Chemotherapy + triptorelin + tibolone (30) 2. Chemotherapy alone (26)	Resumption of menses; monitoring of serum levels of FSH, LH, 17β-E2, and inhibin B during and after chemotherapy; bone mineral density loss monitoring	NM
Blumenfeld et al. (99) (Prospective non-randomized study with historical control)	36 (HD and NHD)	15–40	MOPP/ABV(D), CHOP, C-MOPP, or ABV with or without radiotherapy	Triptorelin depot, 3.75 mg i.m., administered 7–10 days before starting chemotherapy and monthly during chemotherapy treatment, until its conclusion or for a maximum of 6 months	1. Chemotherapy + triptorelin (18) 2. Chemotherapy alone (18)	Resumption of menses and regular cyclic ovarian function; ultrasonographic monitoring of ovarian folliculogenesis and ovulation; monitoring of FSH, LH, estradiol, progesterone, and prolactin levels	up to 4 years (up to 8 years for historical control group)
Blumenfeld et al. (92) (Prospective non-randomized study with concurrent and historical controls)	111 (HD)	15–40	ABVD; MOPP/ABV(D); Standard BEACOPP; Escalated BEACOPP	Triptorelin depot, 3.75 mg i.m., administered 2-7 days before starting chemotherapy and monthly during chemotherapy treatment up to a maximum of 6 months	1. Chemotherapy + Triptorelin (65); 2. Chemotherapy alone (46)	Resumption of menses and cyclic ovarian function; incidence of spontaneous pregnancies; primordial follicle count on both ovaries; FSH, LH, E2 and P levels monitoring	8 years (mean–range 2–15 years)
Pereyra Pacheco et al. (88) (Prospective non-randomized study with historical controls)	16* (HD, NHD, AML)	14.7–20	ICE+BMT; CAVPE+BMT; CCOPP+CAVPE+ESHAP+ ICE+ BMT; CAPVE+BMT; CAVPE+ICE +BMT; CVPP; ABVD; CVPP X 1 + ABVD X 5	Leuprolide depot, 3.75 mg i.m., starting 5-7 days before chemotherapy and monthly during chemotherapy treatment, until 30 days after the end of treatment	1. Chemotherapy (±BMT) + leuprolide (12) 2. Chemotherapy (±BMT) alone (4)*	Resumption of menses and regular cyclic ovarian function; FSH, LH, and estrogens level monitoring; incidence of spontaneous pregnancies; bone density loss monitoring	up to 5 years (up to 6 years in control group)
Blumenfeld et al. (94) (Prospective non-randomized study)	83 (HD, NHL, leukemias and other diseases)	14–40	Conditioning chemotherapy (busulfan and cyclophosphamide; TBI and etoposide; busulfan, cyclophosphamide, fludarabine and antithymocyte globulin; BEAC; or BEAM) before stem cell transplantation	Triptorelin depot, 3.75 mg i.m. 7–14 days before starting gonadotoxic therapy and monthly during chemotherapy	1. Conditioning chemotherapy + triptorelin (47) 2. Conditioning chemotherapy alone (36)	Resumption of menses and cyclic ovarian function; FSH, LH, E2, and P levels monitoring; ultrasonographic monitoring of ovarian antral follicles and stimulated endometrium; spontaneous pregnancies	7 years (median-range 2–13 years) for triptorelin arm and 8 years (median -range 2-13 years) for controls
Blumenfeld et al. (96) (Retrospective case-control study)	474 (HD, NHL, leukemias and other diseases)	14–40	ABVD (± MOPP); BEACOPP/escalated BEACOPP; BMT; CHOP/CVAD	Triptorelin depot, 3.75 mg i.m., administered 7-14 days before starting chemotherapy and monthly during chemotherapy treatment	1. Chemotherapy + triptorelin (286) 2. Chemotherapy alone (188)	Spontaneous pregnancy rate; resumption of menses; FSH, LH, E2, and P levels monitoring; ultrasonographic monitoring of ovarian folliculogenesis and ovulation	up to 25 years (range 2–25 years)
Meli et al. (100) (Retrospective observational study)	36 (ALL; AML; HD; NHL; solid tumors)	11–18	Chemotherapy protocols containing alkylating agents (cyclophosphamide; procarbazine; ifosfamide; carmustine; mitoxantrone; melphalan; busulfan; thiotepa; treosulfan; muphoren) ± radiotherapy or high-dose chemotherapy and HSCT	Triptorelin depot, 3.75 mg i.m. monthly during chemotherapy or 11.25 mg every 3 months, for 3 to 12 months (median, 8 months)	1. Chemotherapy + triptorelin (27)	Resumption of regular spontaneous menstrual cycle; FSH, LH, E2 and P levels monitoring; ultrasonographic visualization of ovarian follicles or corpora lutea; spontaneous pregnancies	7 years from diagnosis (median -range 2-18 years); 5 years from stop therapy (median -range 1–17 years)
Gini et al. (101) (Retrospective and Cross-sectional study)	97 (HD or NHL)	16–50	ABVD or ABVD-like regimens, RCHOP, or VACOP-B ± radiotherapy; second-line regimens followed by HSCT	NM	1. Chemotherapy + oral contraceptives or GnRHa 2. Chemotherapy alone	Resumption of menstrual activity; use of oral contraceptives or GnRHa during chemotherapy; number of pregnancies and offsprings after therapy	NM

*In the study there is another historical control group of premenarchal patient not treated with GnRHa, not mentioned here (n=5).

NM, not mentioned; HD, Hodgkin’s disease; NHL, non-Hodgkin lymphoma; AML, acute myeloid leukemia; ALL, acute lymphoblastic leukemia; ABV, doxorubicin, bleomycin, and vinblastine; ABVD, doxorubicin, bleomycin, vinblastine, dacarbazine; ASCT, autologous stem cell transplantation; BEAC, BCNU, etoposide, cytarabine, cyclophosphamide; BEACOPP, bleomycin, etoposide, adriamycin, cyclophosphamide, vincristine, procarbazine, prednisone; BEAM, BCNU, etoposide, cytarabine, melphalan; BEP, bleomycin, etoposide, cisplatin; BMT, bone marrow transplantation; CAVPE, cyclophosphamide, adriamycin, vincristine, prednisone, etoposide; CCOPP, CCNU (lomustine), cyclophosphamide, vincristine, procarbacine, prednisone; CHOP, cyclophosphamide, adriamycin, vincristine, prednisone; C-MOPP, cyclophosphamide, vincristine, procarbazine, prednisolone; CP, taxol, cisplatin; CVAD, cyclophosphamide, vincristine, doxorubicin, dexamethasone; CVPP, cyclophosphamide, vinblastine, procarbazine, prednisone; ESHAP, etoposide, methylprednisolone, high-dose cytarabine, and cisplatin; HSCT, hematopoietic stem cell transplantation; ICE, ifosfamide, carboplatin, etoposide; MINE, mesna, ifosfamide, mitoxantrone, and etoposide; MOPP, mechloroethamine; vincristine; procarbazine, prednisolone; R-CHOP, rituximab, cyclophosphamide, adriamycin, vincristine, prednisone; RTP, radiotherapy; TBI, total body irradiation; TC, taxol, carboplatin; VAC, vincristine, dactinomycine, cyclophosphamide; VACOP-B, etoposide, doxorubicin, cyclophosphamide, vincristine, prednisone, and bleomycin.

However, such poor outcome could be explained by the fact that almost all patients received at least one prior chemotherapy regimen (median number before HSCT = 2), and 12 patients also received prior local radiation. Therefore, ovarian reserve was probably affected by previous gonadotoxic exposure. Another limitation was the use of a very high dosage of GnRHa. Whereas previous studies used monthly 3.75 mg triptorelin or monthly 3.6 mg goserelin or 11.25 mg leuprolide every 3 months, in this study 22.5 mg leuprolide were administered in 3-month depot injection within 2 months of HSCT. The high doses used in this trial led to intolerable side effects and treatment discontinuation in some patients (104).

Several prospective non-randomized studies have shown the ability of GnRHa to provide a powerful instrument for protection of the ovarian function even in adolescents with hematological malignancies (88, 96, 99, 101, 105).

In a prospective case series with control (88), postpubertal adolescents with normal ovarian function who received monthly leuprolide before and during polychemotherapy for lymphoma, resumed their menstrual cycles and ovulation. After a follow-up of five years, three normal pregnancies were reported. In contrast, patients in the control group had permanent hypergonadotropic amenorrhea.

A long-term follow-up analysis (up to 15 years) of adolescent and young adult with Hodgkin lymphoma co-treated with triptorelin, confirmed the gonadoprotective effect of GnRHa (92). Indeed 96.9% in the GnRHa group resumed ovulation and regular menses, throughout a median follow-up of 8 years (range 2–15), compared with 63% in the control group.

Interestingly, a case report (91, 106) documented four spontaneous pregnancies and two successful deliveries in a patient previously undergoing repeated SCTs and monthly GnRHa co-treatment. SCT almost invariably induces POF owing to higher chemotherapy doses and possible total-body irradiation (107, 108). The estimated odds for spontaneous conception after two SCTs became negligible. These results are highly suggestive that the administration of GnRHa before and during chemotherapy might have minimized the gonadotoxic effects and increased the chance of spontaneous ovulation and successful conception and delivery.

More recently, a prospective, non-randomized study compared the rate of POF after SCT in adolescent and young women receiving GnRHa with gonadotoxic chemotherapy vs chemotherapy alone (94). The study found that GnRHa co-treatment may significantly decrease the POF rate from 82–33% in patients with lymphomas. Moreover, a recent single-center retrospective study on postmenarchal adolescent patients (median age 14, range 11 to 18) showed that GnRHa preserved ovarian function and fertility in adolescents treated for acute lymphoblastic leukemia, acute myeloid leukemia, Hodgkin lymphoma, or other cancers (100). On the last clinical visit, 29 patients (81%) had a regular menstrual cycle, three (8%) oligomenorrhea, and four (11%) amenorrhea. All the four patients with amenorrhea received HSCT. No differences were observed among patients’ disease.

Even these trials confirmed the acceptable safety profile of GnRHa in this setting, with only frequent estrogen deprivation symptoms, reversible upon discontinuation, and bone metabolism alterations not significant for therapies <6 months.

Globally, these clinical data suggest that GnRHa may represents a very useful tool even in post-pubertal adolescents not only for reducing the risk of hypermenorrhea associated with hematologic malignancies or myelosuppressive chemotherapy, but also for preserving ovarian function and fertility, especially when the other established methods of FP (e.g. oocyte cryopreservation) cannot be performed, but also in combination with them in order to increase the odds of success for a specific patient.

At the moment, a phase II/III (NCT02856048), and two phase II (NCT04536467 and NCT03475758) randomized open-label trials including adolescents and pediatric patients are ongoing (**Table 3**).

**Table 3:** Ongoing clinical trials (www.clinicaltrial.gov; update November 2020).

ID	Title	Trial design	Age range (years)	Number of estimated patients (disease)	Arms and interventions	Outcome measures	Follow-up duration
NCT02856048	Co-treatment With GnRH Analogs on the Ovarian Reserve in Young Women Treated With Alkylating Agents for Cancer (PRESOV Study), Sponsor Assistance Publique-Hôpitaux de Paris	Phase II/III randomized open-label	12–25	160 (Ewing Sarcoma, Osteosarcoma, Lymphoma)	1. Triptorelin 3 mg i.m. every 28 ± 3 days + Chemotherapy with alkylating agents at an intermediate ovarian toxicity risk* 2. Chemotherapy alone	Variation in AMH serum levels at 24 months; AFC on ultrasound at 24 months; delay of resumption of menses; AMH, FSH, estradiol levels monitoring; pregnancy rate at 3 years; GnRH-related AEs; change in BMD at 12 and 36 months	3 years
NCT04536467 (actual completion date June 1th 2020)	Prevention of Chemotherapy-Induced Ovarian Failure With Goserelin in Premenopausal Lymphoma Patients, Sponsor Beni-Suef University	Phase II randomized open-label	17–40	34 (Lymphoma)	1. Goserelin 3.6 mg s.c. 28 ± 3 days + standard chemotherapy 2. Standard chemotherapy alone	FSH and E2 levels at 6 months; overall response rate in lymphoma patients** at 6 months; GnRH-related AEs	6 months
NCT03475758	Goserelin for Ovarian Protection in Premenopausal Patients Receiving Cyclophosphamide, Sponsor Assiut University	Phase II randomized open-label	NR (Child and adult)	100 (Cancer patients)	1. Goserelin 3.6 mg s.c. every 4 weeks + cyclophosphamide containing chemotherapy 2. Cyclophosphamide containing chemotherapy alone	Rate of ovarian failure at 1 year (assessed by hormonal profile – FSH, LH, estradiol – every 6 months)	1 year

*Cyclophosphamide 6 g/m2, Ifosfamide 50 g/m2, Procarbazine 4 g/m2, Lomustine 350 mg/m2 or Melphalan 140 mg/m2 or a combination of these drugs; ** determined by tumor assessments from radiological tests (CT scan, MRI, Positron-emission tomography or physical examinations); AFC, Antral follicular count; AMH, Anti-Mullerian hormone; BMD, Bone Mass Density; FSH, Follicle-stimulating hormone; NR, not reported.

## The Regulatory Perspective: Off-Label Use

To date the three analogues (triptorelin, goserelin, leuprolide) are authorized for various therapeutic indications, such as cancer, endometriosis, uterine fibroids, and precocious puberty. Therefore, the prescription for preventing the risk of POF is typically an off-label use, defined as the use of a medicinal product “*for a medical purpose not in accordance with the authorized product information*” (109). European Union. Study on off-label use of medicinal products in the European Union. Available at: https://ec.europa.eu/health/sites/health/files. Off-label is not regulated at the European level, but specific national measures have been adopted (109, 110). In general, this use is not reimbursable excluding selected cases defined by law. For example, the *Recommandations Temporaires d’Utilisation* (RTU) provide coverage of recognized off-label treatment by France Health Insurance (111).

Moreover, the Italian national health system (NHS) reimburses an off-label use according to Law 648/1996 based on results from at least phase II trials (112). The inclusion into the 648/1996 list of reimbursable drugs ensures a nationwide access according to criteria for appropriate use and monitoring defined by the AIFA Scientific Committee in the light of clinical evidence.

From 2016 this Italian law allows to reimburse GnRHa for the preservation of ovarian function in pre-menopausal women at risk of premature and permanent menopause following chemotherapy treatment (29). The Italian regulatory authority defined the eligibility criteria, including age (>18 and <43) and lack of adequate alternative options. Thus, currently the use in the post-pubertal age is not approved and falls within the Italian Law 94/1998 (113), by which physicians can perform off-label prescriptions (not covered by the NHS) but in individual and exceptional cases.

This represents to date a limit for the treatment of this population that could be overcome if the eligibility criteria of Law 648/96 will be modified in order to include even pediatric patients.

Currently, to the best of our knowledge no other countries gave a nation-wide approval for this systematic off-label use.

## Conclusion

Ovarian failure following chemotherapy represents an adverse event with an important impact on health and quality of life of survivors. Oocyte and tissue cryopreservation are the main options for fertility preservation. However, these techniques cannot be performed in all patients in all health-care facilities. Pharmacological treatment with GnRHa is a non-invasive and well-tolerated alternative, which can be offered to all subjects if cryopreservation is not feasible due to clinical or logistic issues. Moreover, combining several methods may increase the odds of success of fertility preservation in eligible patients. The available evidence demonstrates a significant advantage for the survivors who received the GnRHa in the long-term maintenance of ovarian function and preservation of fertility. Italy has officially recognized the off-label use of GnRHa in women at risk of premature and permanent menopause following chemotherapy. However, fertility preservation still represents an unmet medical need in adolescents, especially in those who cannot access to other therapeutic options.

## Author Contributions

SB and LG wrote the first draft of the manuscript. FD checked and revised the draft manuscript. All authors contributed to the article and approved the submitted version.

## Funding

The open access publication fees will be funded within the AIFA pharmacovigilance grant, project ADR-648 “Monitoring of safety and efficacy of drugs prescribed under Law 648/96”.

## Conflict of Interest

The authors declare that the research was conducted in the absence of any commercial or financial relationships that could be construed as a potential conflict of interest.
