# Supplementary material for: Fertility Preservation in Female Pediatric Patients With Cancer: A Clinical and Regulatory Issue
Source: Front Oncol. 2021 Mar 9;11:641450. doi: 10.3389/fonc.2021.641450 (PMC8008167; doi:10.3389/fonc.2021.641450)
Supplement: Supplementary file 1 [file Table_1.docx]

**Supplementary 1:** randomized clinical trials (RCTs) assessing the use of GnRHa in the prevention of chemotherapy damage in pre-menopausal women with breast cancer

| **Author**  **(Study Design)** | **Number of Patients**  **(Disease)** | **Age range**  **(years)** | **Chemotherapy protocol** | **GnRHa (dosage/posology-duration)** | **Treatment arms (patients per arm)** | **Outcome measures** | **Follow-up duration** |
| --- | --- | --- | --- | --- | --- | --- | --- |
| Badawi et al. (2009) [58]  (Prospective, randomized clinical trial) | 80  (breast cancer) | 18 - 40 | FAC regimen  (a combination of  5-Fluorouracil 500 mg/m^2^ i.v., Doxorubicin 500 mg/m^2^ i.v., and Cyclophosphamide 500 mg/m^2^ i.v.) every 6–8 weeks for 6 cycles | Goserelin at a dose of 3.6 mg subcutaneously every 28 days for 6 months | 1. Chemotherapy + goserelin (40)  2. Chemotherapy alone (40) | Resumption of ovulation and menses and hormonal profile | 8 months |
| Sverrisdottir et al. (2009) [59]  (open label, randomized clinical trial) | 260  (breast cancer) | 29 - 54 | Adjuvant CMF chemotherapy (six cycles of cyclophosphamide 600 mg/m^2^, methotrexate 40 mg/m^2^ and 5-fluorouracil 600 mg/m^2^ i.v. on days 1 and 8, every 28 days), in addition to endocrine therapy | Goserelin 3.6 mg subcutaneously every 28 days | 1. Goserelin (63)  2. Goserelin + tamoxifen (74)  3. Tamoxifen alone (60)  4. No endocrine treatment (63) | Resumption of menses | 36 months |
| Del Mastro et al. (2011) [60] Lambertini et al. (2015) [61]  (parallel, randomized, open-label, phase 3 superiority trial) | 282  (breast cancer) | 24 - 45 | Adjuvant or neoadjuvant treatment with anthracycline-based, anthracycline plus taxane–based, or CMF-based (100 mg/m^2^ of oral cyclophosphamide on days 1-14 or 600 mg/m^2^ of i.v. cyclophosphamide on days 1 and 8; 40 mg/m^2^ of methotrexate on days 1 and 8; and 600 mg/m^2^ of fluorouracil on days 1 and 8) chemotherapy. | Triptorelin 3.75 mg at least 1 week before starting chemotherapy and then every 4 weeks for the duration of the treatment | 1. Chemotherapy + triptorelin (148)  2. Chemotherapy alone (133) | Resumption of menstrual activity and postmenopausal levels of follicle stimulating hormone (FSH) | 12 months |
| Song et al. (2013) [62]  (prospective randomized phase II study) | 220  (breast cancer) | 26 - 45 | 1) 600 mg/m^2^ cyclophosphamide and 60 mg/m^2^ doxorubicin (CA) every 3 weeks for six cycles and  2) 600 mg/m^2^ cyclophosphamide, 60 mg/m^2^ doxorubicin and 175 mg/m^2^ paclitaxel (CA + Taxol) every 3 weeks for four cycles | Leuprolide acetate 3.75 mg every 4 weeks | 1. Chemotherapy + leuprolide acetate (89)  2. Chemotherapy alone (94) | Menstrual activity resumption, FSH and E2 levels | 12 months |
| Karimi-Zarchi et al. (2014) [64] (randomized clinical trial) | 42  (breast cancer) | 25 - 45 | Cyclophosphamide (600 mg/m^2^), adriamycin (60 mg/m^2^), and taxoter (75 mg/m^2^) chemotherapy regimens | Triptorelin embonate 3.75 mg i.m. every 28 days for 6 months | 1. Chemotherapy + triptorelin embonate (21);  2. Chemotherapy alone (21) | Resumption of ovulation and menses and hormonal profile | 6 months |
| Moore et al. (2015) [65], Moore et al. (2019) [66] (phase 3 trial randomized study) | 218  (breast cancer) | 25 - 49 | Adjuvant or neoadjuvant cyclophosphamide-containing chemotherapy | Goserelin 3.6 mg subcutaneously every 4 weeks beginning 1 week before the initial chemotherapy dose and continued to within 2 weeks before or after the final chemotherapy dose | 1. Chemotherapy + goserelin (105)  2. Chemotherapy alone (113) | Resumption of menses and regular cyclic ovarian function; hormonal level monitoring; pregnancies | 5 years |
| Leonard et al. (2017) [67]  (Prospective randomized study) | 227  (breast cancer) | 24 - 51 | Six to eight cycles of cyclophosphamide and/or anthracycline-containing regimens with or without a taxane | 3.6 mg goserelin implant starting at least 1 week (preferably 2 weeks) prior to the start of the chemotherapy treatment, and continuing 3–4 weekly until the end of the chemotherapy treatment | 1. Chemotherapy + goserelin (106)  2. Chemotherapy alone (121) | Resumption of menses and cyclic ovarian function; hormonal level. | 5 years |
| Zhang et al. (2018) [68]  (phase 3, open-label, parallel, randomized controlled trial) | 216  (breast cancer) | 18 - 45 | Chemotherapy + tamoxifen +/-trastuzumab | Goserelin 3.6 mg s.c. every 4 weeks, or leuprorelin 11.25 mg s.c. every 12 weeks, starting 1 week before the initial chemotherapy and continued at least for 2 years | 1. Chemotherapy + goserelin or leuprorelin (108)  2. Chemotherapy alone (108) | Resumption of menses and cyclic ovarian function; hormonal level. | 56.9 months (range 49.5–72.4 months) |

**Supplementary 2:** results of clinical trials involving paediatric patients

|  | **Preserved Ovarian function N (%)** | | **Amenorrhea/POF N (%)** | | **N. of pregnant patients**  **(N. of pregnancies)** | |
| --- | --- | --- | --- | --- | --- | --- |
|  | **Study** | **Control** | **Study** | **Control** | **Study** | **Control** |
| Gilani et al. (2007) [74] | 15/15 (100%) | 10/15 (67%) | 0 | 5/15 (33%) | /** | /** |
| Cheng et al. (2012) [92] | 7/44 (16%) | /* | 37/44 (84%) | /* | /** | /* |
| Castelo-Branco et al. (2007) [81] | 27/30 (90%) | 6/26 (23%) | 3/30 (10%) | 20/26 (77%) | 1 (1) | NM |
| Blumenfeld et al. (1996) [94] | 15/16 (93.7%) | 7/18 (39%) | 1/16 (6.3%) | 11/18 (61%) | 3 (4) | NM |
| Blumenfeld et al. (2008) [84] | 63/65 (96.9%) | 29/46 (63%) | 2/65 (3.1%) | 17/36 (37%) | 19 (26) | 12 (20) |
| Pereyra Pacheco et al. (2001) [80] | 12/12 (100%) | 0 | 0 | 4/4 (100%) | 2 (3) | NM |
| Blumenfeld et al. (2012) [86] | 18/47 (38.3%) | 4/36 (11.1%) | 29/47 (61.7%) | 32/36 (88.9%) | 2 (6) | 0 |
| Blumenfeld et al. (2015) [88] | 127/146 (87%) | 35/71 (49%) | 19/146 (13%) | 36/71 (51%) | 85/122 (169) | 28/66 (49) |
| Meli et al. (2018) [100] | 29/36 (81%) | /* | 7/36 (19%) | /* | 5 (8) | /* |

NM = not mentioned; * only one study group; ** data not registered/not included in outcome measures.
